# AttOmics: attention-based architecture for diagnosis and prognosis from omics data

**DOI:** 10.1093/bioinformatics/btad232

**Published:** 2023-06-30

**Authors:** Aurélien Beaude, Milad Rafiee Vahid, Franck Augé, Farida Zehraoui, Blaise Hanczar

**Affiliations:** IBISC, Université Paris-Saclay, Univ Evry, 23 Boulevard de France, Evry-Courcouronnes 91020, France; Artificial Intelligence & Deep Analytics, Omics Data Science, Sanofi R&D Data and Data Science, 1 Av. Pierre Brossolette, Chilly-Mazarin 91385, France; Sanofi R&D Data and Data Science, Artificial Intelligence & Deep Analytics, Omics Data Science, 450 Water Street, Cambridge, MA 02142, United States; Artificial Intelligence & Deep Analytics, Omics Data Science, Sanofi R&D Data and Data Science, 1 Av. Pierre Brossolette, Chilly-Mazarin 91385, France; IBISC, Université Paris-Saclay, Univ Evry, 23 Boulevard de France, Evry-Courcouronnes 91020, France; IBISC, Université Paris-Saclay, Univ Evry, 23 Boulevard de France, Evry-Courcouronnes 91020, France

## Abstract

**Motivation:**

The increasing availability of high-throughput omics data allows for considering a new medicine centered on individual patients. Precision medicine relies on exploiting these high-throughput data with machine-learning models, especially the ones based on deep-learning approaches, to improve diagnosis. Due to the high-dimensional small-sample nature of omics data, current deep-learning models end up with many parameters and have to be fitted with a limited training set. Furthermore, interactions between molecular entities inside an omics profile are not patient specific but are the same for all patients.

**Results:**

In this article, we propose AttOmics, a new deep-learning architecture based on the self-attention mechanism. First, we decompose each omics profile into a set of groups, where each group contains related features. Then, by applying the self-attention mechanism to the set of groups, we can capture the different interactions specific to a patient. The results of different experiments carried out in this article show that our model can accurately predict the phenotype of a patient with fewer parameters than deep neural networks. Visualizing the attention maps can provide new insights into the essential groups for a particular phenotype.

**Availability and implementation:**

The code and data are available at https://forge.ibisc.univ-evry.fr/abeaude/AttOmics. TCGA data can be downloaded from the Genomic Data Commons Data Portal.

## 1 Introduction

The disruption of different biological processes (BPs) can negatively affect an organism and lead to a disease state. Early diagnosis plays an important role in precision medicine in order to improve clinical decision-making. The development of high-throughput methods influenced precision medicine by enabling easy access to a large amount of biological information for each patient, known as omics profile. Omics profiles are high-dimensional complex signatures resulting from interactions of many molecular entities. The first common step in most machine learning approaches used in precision medicine is a feature selection procedure that reduces the data’s size to construct a classifier from a single-omics (Kourou et al. 2015). As the selection procedure is decoupled from the prediction task, only selected features are used for downstream predictions (Liu et al. 2019). Using only selected features limit the model’s capacity to extract hidden information from the omitted features. Deep learning, on the contrary, can extract and exploit the complete information from all features and their interactions. This characteristic may be useful to achieve better-predicting performances.

Following the recent successes of deep learning in computer vision or natural language processing (LeCun et al. 2015), different deep learning architectures were successfully applied to omics data. It allows for a high level of abstraction of features with nonlinear modeling and can handle complex dependencies in data to create informative representations. Assuming that omics data does not have any particular structure, unlike images or texts, multilayer perceptrons (MLP) were used to perform predictions (Yu et al. 2021) and autoencoders (AE) for dimension reduction (Gore and Azad 2022). Other approaches tried to integrate a structure in the model by embedding biological knowledge and applying convolutional neural networks (CNN) (Elbashir et al. 2019) or graph neural networks (GNN) (Ramirez et al. 2020).

Cellular functions are governed by the combined action of multiple molecular entities which are specific to a patient. The expression of one gene may impact the expression of other genes differently in different patients. With classical deep learning approaches, these interactions which are learned during training, are assumed to be identical for all patients in the inference phase. It would be more beneficial to compute feature interactions that are specific to each patient. Self-attention can be used to improve the representation of the features vector by incorporating dynamically computed relationships between elements of the vector. It has been shown that the transformer architecture’s promising results extensively rely on attention mechanisms (Vaswani et al. 2017).

Here, we propose a new method based on the self-attention mechanism to capture interactions between different molecular entities in order to predict the phenotype of patients, e.g. cancer types or the risk of death from omics data. Using a self-attention mechanism allows the model to capture feature interactions specific to each patient dynamically. Applying self-attention on high dimensional vectors such as omics profiles is challenging as the self-attention memory requirements scale quadratically with the number of elements. To overcome this problem, we propose to consider groups of features and apply self-attention to these groups. The architecture was tested on three different omics data: gene (mRNA) expression, methylation (DNAm), and micro-RNA (miRNA) expression and was compared with state-of-the-art deep learning-based methods. The results show that our proposed architecture better considers feature interactions in omics data and improves the model performance.

## 2 Related work

Different deep learning approaches have already been tested on omics data: CNN, GNN, MLP, AE, and variational autoencoder (VAE). Hanczar et al. (2022) showed that MLP outperforms the classic machine learning methods on large gene expression datasets. Yu et al. explored different MLP architectures by varying the number of neurons in each layer and the number of layers. They showed that wider networks perform better than deeper ones. DeepCC (Gao et al. 2019) applies an MLP on biologically informed features by transforming gene expression data into a functional spectrum, i.e. a list of enrichment scores calculated by gene set enrichment analysis. There are also other approaches that included biological knowledge in the design of the neural networks by restricting connections between neurons to known biological relations, such as Gene Ontology (GO) (Bourgeais et al. 2021) or REACTOME (Hao et al. 2021).

A VAE unsupervised training has been used as a pre-training for an MLP classifier (Levy et al. 2020), and the VAE latent space has also been directly passed to a classifier (Wang and Wang 2018). Another approach constrained the latent space to learn relevant features for the classification by end-to-end training a network on both the unsupervised and supervised tasks (Gore and Azad 2022).

Promising results of CNN in computer vision inspired its application in precision medicine. Different strategies were developed to create a 2D image from an expression vector. Some approaches reshaped a 1D omics vector into a 2D image to exploit the capacity of CNN architectures to extract relevant visual patterns (Elbashir et al. 2019; Mostavi et al. 2020; Rukhsar et al. 2021). Ma and Zhang (2019) created an image by transforming an expression vector into a tree map based on the Kyoto encyclopedia of genes and genomes Brite structure. Instead of forcing a 2D representation, 1D convolution has been applied to an ordered expression vector (Mostavi et al. 2020; Zhao et al. 2020). In Zhao et al. (2020), the expression vector was reordered according to the chromosomal locations before applying a 1D inception architecture. Expression profiles have also been represented as graphs to represent the interactions of the different molecular entities. A graph convolutional network based on a co-expression network or a protein–protein interaction (PPI) network was used to predict cancer types from gene expression (Ramirez et al. 2020). Ramirez et al. (2021)  explored the combination of a co-expression (CoExp) graph and a graph constructed from the GeneMania database.

In Levy et al. (2021), an architecture inspired by the capsule network was used to predict the central nervous system tumors subtype. Methylation features have been grouped into capsules to create context-specific embeddings, and dynamic routing was then applied to make a prediction.

Deep learning architectures using attention mechanisms have been little explored for the application of omics data. Some approaches helped the network to focus on relevant genes for the predicted phenotype by computing feature importance scores with a small neural network (Beykikhoshk et al. 2020; Lee 2022). Those methods were inspired by the attention mechanism but did not use the original dot product self-attention (Vaswani et al. 2017) layers directly on the omics data. Computing self-attention on a high dimensional vector is hardware limited as memory requirements scale quadratically with the number of elements. The Gene transformer (Khan and Lee 2021) was the first architecture to apply self-attention to mRNA data. The authors proposed to use 1D convolution layers combined with maximum pooling to reduce the dimension of the gene expression vector. Using a pooling layer is equivalent to a dimension reduction that does not consider all possible feature interactions.

In our approach, AttOmics, we propose to embed groups of features in a lower dimension by considering all interactions inside this group and a new way of applying self-attention (Vaswani et al. 2017) to omics data that takes into account inter-group interactions. AttOmics can be applied to vectors of various sizes, and consequently, detect feature interactions in different omics data.

## 3 Model architecture

### 3.1 Architecture details

**Figure 1.**
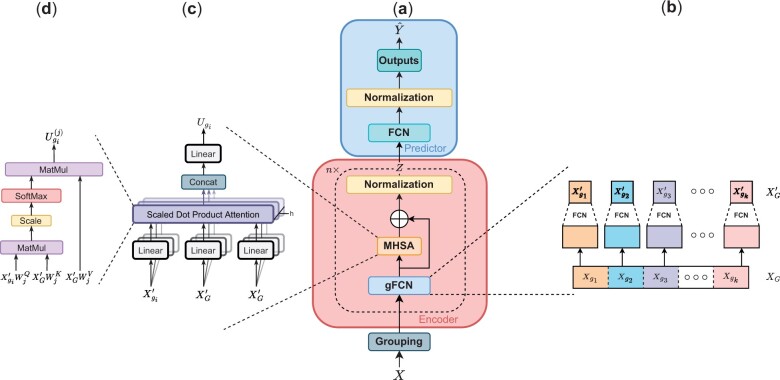
The AttOmics architecture is composed of a grouping module, an encoder and a predictor (a). The grouping module transforms the input features into a set of different groups. In each of the *n* encoder blocks, each group is projected into a lower dimensional space (b). Interactions between the different groups is computed with the MHSA (c). In each of the *h* heads of the MHSA, a scaled dot product attention is computed between the different groups (d). A residual connection is added around the MHSA before applying a normalization. The new representation obtained with the encoder is transmitted to the predictor, an FCN followed by a normalization.

The model includes a grouping module and an encoder followed by a predictor, illustrated in Fig. 1a. Instead of considering each feature individually, features are divided into different groups. The encoder is a stack of *n* blocks used to construct a new representation of the inputs. Each block is formed of a grouped fully connected network (gFCN) module where each group is projected into a lower dimension with a fully connected network (FCN). Segregating features in groups restrict the potential interactions between features to the ones inside the same group. Multihead self-attention (MHSA) is applied to the set of groups to recover all possible interactions between groups. Around the self-attention block, a residual connection is added before applying a normalization. The encoder output is transmitted to an FCN used as the predictor.

#### 3.1.1 Grouped FCN

Let $X\in R^{p}$ be a training example where *p* is the number of features and *Y* is the associated label. The training example *X* is split into groups according to a grouping strategy (see Section 3.2), $X_{G}={\left\{X_{g_{i}}\right\}}_{1\leq i\leq k}$, where *k* is the number of groups. For each group $X_{g_{i}}$, a group embedding is independently computed as $X_{g_{i}}\prime$ by projecting it into an *s*-dimensional space with an FCN, a succession of fully connected layers (FCL) (Fig. 1b).

Each FCL is the composition of an affine transformation of its inputs with a rectified linear unit (ReLU) activation function:


FCL(x)=ReLU(Wx+b)=max(0,Wx+b)

After processing each group $X_{g_{i}}$ by the successive FCL, we obtain the set of group embeddings $X\prime_{G}$: where $X_{g_{i}}\prime =\text{FCN}\left(X_{g_{i}}\right)$.


$$X_{G}^{\prime }={\left\{X_{g_{i}}\prime \in R^{s}\right\}}_{1\leq i\leq k},$$

Each group projection is only computed using elements from the same group. To create a representation of the expression vector based on all possible interactions, MHSA is then applied to $X\prime_{G}$.

#### 3.1.2 Multihead self-attention

MHSA is applied to construct a new representation of the groups, $U={\left\{U_{g_{i}}\right\}}_{1\leq i\leq k}$, by allowing them to interact with each other (Fig. 1c).

MHSA is performed with *h* different heads to learn different types of interactions. For each head *j*, self-attention is applied to each group *g_i_* (1≤i≤k), in order to obtain: $U^{\left(j\right)}={\left\{U_{g_{i}}^{\left(j\right)}\in R^{l}\right\}}_{1\leq i\leq k}$, where $l=\frac{s}{h}\in N$.

$U_{g_{i}}^{\left(j\right)}$ is defined by where $A_{g_{i}}^{\left(j\right)}$ is the attention vector computed by the usual dot product attention (Fig. 1d) (Vaswani et al. 2017):


$$U_{g_{i}}^{\left(j\right)}=A_{g_{i}}^{\left(j\right)}\cdot {\left[X_{g_{1}}\prime \cdot W_{j}^{V},\ldots ,X_{g_{k}}\prime \cdot W_{j}^{V}\right]}^{T},$$

$$\begin{array}{c} A_{g_{i}}^{\left(j\right)}=\text{softmax}\left(\left[A_{g_{i},g_{1}}^{\left(j\right)},\ldots ,A_{g_{i},g_{k}}^{\left(j\right)}\right]\right),\\ A_{g_{i},g_{k}}^{\left(j\right)}=\frac{{\left(X_{g_{i}}\prime \cdot W_{j}^{Q}\right)}^{T}\cdot \left(X_{g_{k}}\prime \cdot W_{j}^{K}\right)}{\sqrt{s}}. \end{array}$$

Projection matrix $W_{j}^{Q}$ (respectively $W_{j}^{K}$ and $W_{j}^{V}$) maps the group $X_{g_{i}}\prime$, from an *s*-dimensional space to an *l*-dimensional space. In the transformers formulation $X_{g_{i}}\prime \cdot W_{j}^{Q},\;X_{g_{k}}\prime \cdot W_{j}^{K}$, and $X_{g_{i}}\prime \cdot W_{j}^{V}$ are called, query, key, and value, respectively.

Each element of $U_{g_{i}}$ is obtained by concatenating the representation of all groups in the different heads and projecting each group to an *s*-dimensional space using a projection matrix $W^{O}\in R^{s\times s}$ as:


$$U_{g_{i}}=\text{concat}\left(U_{g_{i}}^{\left(1\right)},\ldots ,U_{g_{i}}^{\left(h\right)}\right)\cdot W^{O},$$

#### 3.1.3 Residual connection and normalization

The value of $X_{g_{i}}\prime$ is added to $U_{g_{i}}$, through a residual connection to prevent vanishing gradients.

The last step in the encoder module consists of applying a normalization to obtain the final representation $Z_{g_{i}}$ of group *g_i_* defined as


$$Z_{g_{i}}=\text{Norm}\left(X_{g_{i}}\prime +U_{g_{i}}\right).$$

The output of an encoder block is $Z={\left\{Z_{g_{i}}\in R^{s}\right\}}_{1\leq i\leq k}$, which is a representation of the groups capturing their interactions.

#### 3.1.4 Prediction module

The vectors $Z_{g_{i}}$ are concatenated into a new vector $Z\prime \in R^{ks}$. The output of the encoder Z′ is then fed to a FCN followed by a normalization layer to predict the cancer type or the prognosis $\hat{Y}$ (Fig. 1a).

For classification tasks, the output layer has one neuron per class, and a softmax activation function is applied to get the probability vector $P={\left[p_{c}\right]}_{1\leq c\leq M}$, where *M* denotes the number of classes. For the survival analysis, the output is a single neuron with a linear activation function.

### 3.2 Grouping strategies

The AttOmics architecture can be applied to any group specification. We explore different grouping strategies such as random groups, groups obtained with clustering, groups based on biological information like the GO (Gene Ontology Consortium 2021) or the hallmarks collection available in MSigDB (Liberzon et al. 2015).

#### 3.2.1 Random

With the random strategy, groups are formed by randomly sampling the input features in groups of similar sizes, *p*/*k*.

#### 3.2.2 Gene ontology

We used BP gene ontology as it groups different molecular activities in a shared process which are more likely linked to the same cancer phenotype. To avoid possible problems with selecting the GO terms (i.e. groups) of interest, we restrict ourselves to terms available in GO slims.

Inside the BP slim ontology, a gene can belong to more than one group; on average, they belong to two groups. Before applying self-attention, each group must be projected into the same dimensional space. Each group is projected with a different number of layers to have the same reduction ratio across different groups. This grouping strategy can only be applied to mRNA data.

#### 3.2.3 Hallmarks

In the MSigDB hallmarks collection (Liberzon et al. 2015), there are 50 groups. Each one represents a well-defined BP. Each group is projected with a different number of layers to ensure identical reduction ratio across different groups. This grouping strategy can only be applied to mRNA data.

#### 3.2.4 Clustering

The clustering strategy groups features based on their expression levels. Traditional clustering methods, like *K*-Means or hierarchical clustering, can return sets of highly unbalanced clusters that may negatively affect the efficiency of our model. Large groups would require many parameters to be projected into a space with a dimension lower than the smallest group. Group unbalances would also imply a high compression of larger groups and almost no compression for the smallest group. To prevent this, we used constrained K-means clustering to ensure comparable group sizes (Bradley et al. 2000).

### 3.3 Model training

For classification problems, our model is trained end-to-end with a weighted cross-entropy loss to account for class imbalance: where *w_c_* denotes the weight (inversely proportional to the size) of class c∈{1,…,M} and *θ* the model parameters.


$$L(\theta )=-\sum_{c=1}^{M}w_{c}Y_{c}\;\text{log}\;\left(p_{c}\right),$$

For survival analysis, our model is end-to-end trained with a partial log-likelihood loss, as proposed in DeepSurv (Katzman et al. 2018): where *δ_i_* specifies if the event occurred for patient *i*, *T_i_* represents the time associated to the event and $N_{\delta_{i}=1}$ is the number of patients for which the event occurred ($\delta_{i}=1$). $\eta_{i}=e^{{\hat{Y}}_{i}}$ is the predicted risk for patient *i*. $R(T_{i})=\{j:T_{j}>T_{i}\}$ is the risk set, the set of patients who are still at risk of death at time *T_i_*.


$$L(\theta )=\frac{1}{N_{\delta_{i}=1}}\sum_{i:\delta_{i}=1}\left({\hat{Y}}_{i}-\text{log}\;\sum_{j\in R(T_{i})}\eta_{j}\right),$$

## 4 Experiments

### 4.1 Data

TCGA data were used to evaluate our proposed approach AttOmics. We collected DNA methylation, gene expression, and miRNA expression data for 8416 patients of 19 different cancers and 361 normal samples from the GDC Data Portal (https://portal.gdc.cancer.gov/). FFPE samples and bad replicates were removed according to TCGA consortium recommendation. Methylation data was restricted to the probes common to both HumanMethylation27 and HumanMethylation450 platforms. No feature selection was applied, and data were standardized to a zero mean and unit variance.

Patients with incorrect survival information were removed: 8349 patients were available for survival prediction. A total of 70% of the data are used as a training set, 15% forms the validation set, and the remaining 15% forms the test set while preserving the proportion of each cancer.

The training set is used to perform two predicting tasks: phenotype prediction, 19 different cancers and normal, and survival risk prediction.

### 4.2 Comparative study

For a comprehensive and comparative evaluation, we choose three deep learning architectures for comparison: CNN, GNN, and MLP.

For the CNN (CNN1d), we ordered features based on their position on the genome, then used a 1D convolution, followed by a ReLU activation and a maximum pooling. For the GNN architecture, two graphs were used: PPI (GNN—PPI) and co-expression (GNN—CoExp) graphs. The PPI graph is based on data available in the STRING database (Szklarczyk et al. 2020) and was constructed by retaining only high-confidence links: edges with a score higher than 700. The CoExp graph was constructed similarly to Ramirez et al. (2020). The Spearman correlation matrix between gene expressions was computed. If the correlation was higher than a threshold and the associated *P*-value was lower than .05, then an edge between the two features was added to the graph. For mRNA and miRNA, the correlation threshold was set to 0.6. For DNAm, a 0.7 correlation threshold was used. Self-loops were not considered in the graph construction, and isolated nodes were removed. The PPI graph and the CoExp graph for mRNA have 9384 genes in common. Each graph is described in the Supplementary Table S2. MLP architecture has two hidden layers with ReLU activation and makes use of batch normalization. We also consider three state-of-the-art non-deep-learning models for comparison: support vector machine (SVM), random forest (RF), and extreme gradient boosting (XGBoost). For the non-deep-learning approaches, the 2000 most discriminative features are selected with a *t*-test-based selection.

The hyperparameters of each approach are tuned on each omics data with a random search to achieve the best performances. The different values tested for each parameter are defined in the Supplementary Table S3. For each hyperparameter at each search iteration, a value is randomly drawn from the defined range. A model is constructed using these parameters, trained on the training set, and evaluated on the validation set. The selected hyper-parameters for each architecture are presented in the Supplementary Table S5.

AttOmics is trained end-to-end using the Adam optimizer with a learning rate of 0.0001 and a batch size of 512. The maximum number of epochs was set to 100. An early stopping strategy is deployed to avoid over-fitting with a patience of 8 and a delta of 0.001 on the validation metric between two epochs.

For the classification task, models were evaluated with the error rate. Prognosis prediction is evaluated with the concordance index (Harrell et al. 1996). It estimates that for a pair of individuals, the predicted risks, *η*, are concordant with their actual survival times.


$$C-\text{Index}=\frac{\sum_{i,j}1_{T_{j}<T_{i}}1_{\eta_{j}>\eta_{i}}\delta_{j}}{\sum_{i,j}1_{T_{j}<T_{i}}\delta_{j}}$$

A C-Index=0.5 represents a random prediction and C-Index=1 corresponds to a perfect ranking. Results for the prognosis task are presented in the Supplementary Material.

## 5 Results

### 5.1 Hyperparameters choice

**Figure 2.**
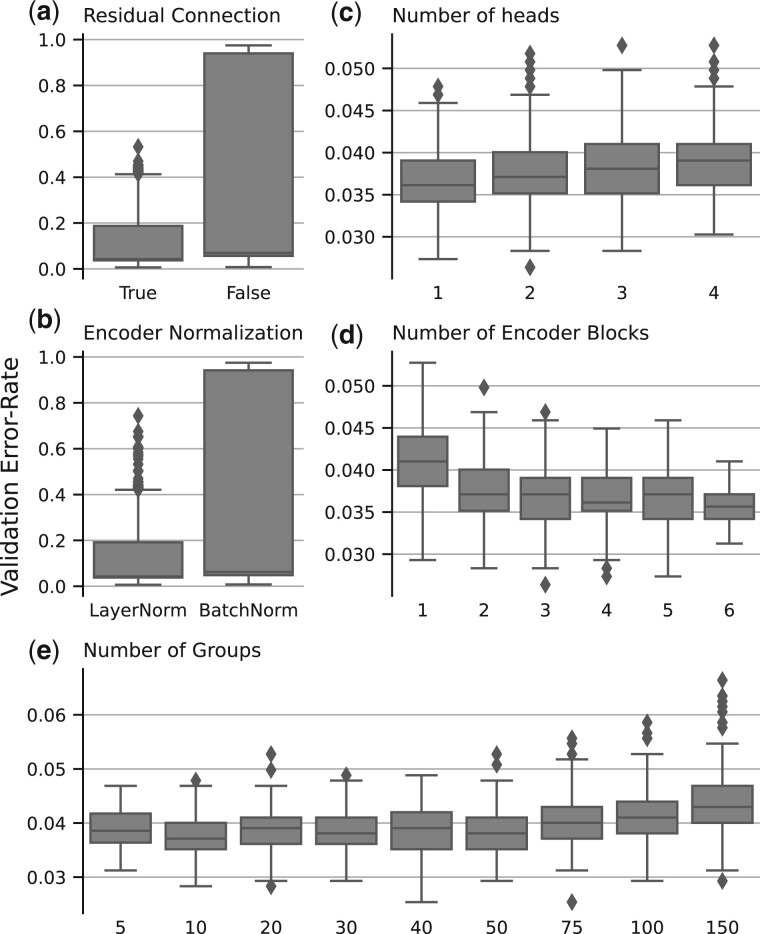
Results of the random search for mRNA data with the clustering grouping strategy.

We investigate the impact of the main hyperparameters on the model error rate by applying a random search procedure. For each hyper-parameter, a random value is drawn from a set of predefined possible values. A model is trained with selected hyperparameters on the training dataset. For each grouping strategy, 1500 models are trained. The performance metrics reported here are estimated on the validation set. The results for the main hyperparameters of this experiment are presented in Fig. 2. The performance obtained for each tested value is represented with a boxplot.

The encoder’s residual connection and the choice of normalization type greatly impact the performances. Adding a residual connection in an encoder block significantly impacts the model’s performance. It stabilizes the model performances (Fig. 2a). We explore two types of normalization in the encoder: layer normalization (LayerNorm) and batch normalization (BatchNorm). Layer normalization gives better and more stable results (Fig. 2b). The different hyperparameters which control the model architecture impact the performances differently. A sufficient number of encoder blocks is required to achieve the best performances. There is an 11% error rate improvement from 1 to 3 blocks. Beyond three blocks, there is no error rate decrease (Fig. 2d). The number of heads used in the MHSA layer has no significant impact on the validation error rate. There is only a 0.002 mean validation error rate difference between 1 and 4 heads (Fig. 2c).

Another important hyperparameter of the architecture is the number of groups (Fig. 2e). An increase in the number of groups impacts the model performances. There is a 10% increase in the error rate between 10 and 100 groups. In the range of 10–50 groups, the impact on the performances is limited. The maximum mean error rate difference observed is 0.004, which is less than 1% of variation. Increasing the number of groups also impacts the model complexity as self-attention scales quadratically with the number of groups.

The selected hyperparameters for each grouping strategy and each omics data are presented in the Supplementary Table S4.

### 5.2 Comparison with state of the art

Despite the broad adoption of high throughput methods in personalized medicine, the availability of omics data from cancer patients remains limited. We explore the impact of the training database size on the performances of AttOmics and other deep-learning architectures by training the different models on a subset of the training set. The different subsets are created by randomly sampling 10%, 30%, 50%, and 70% of the training set while preserving class proportions. To prevent data leakage, structures computed from the training set, like CoExp graphs or clustered grouping, are recomputed with the selected subset. For each subset, five models are trained. The reported performance metrics are estimated on the test set.

**Figure 3.**
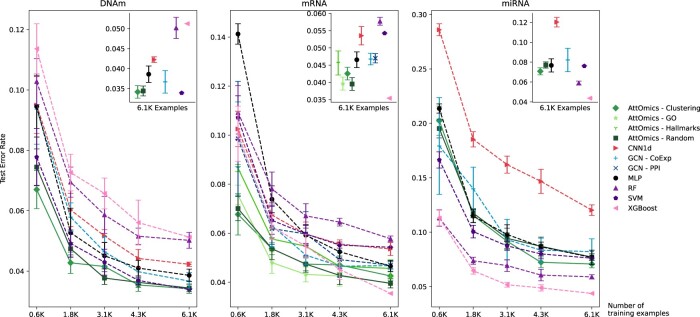
Error rate on the test set according to the size of the training set.

Figure 3 shows the average and standard deviation of the error rate on the cancer-type classification task according to the training set size for all tested methods. A Wilcoxon test is used to assess the significance of the results, *P*-values are corrected for multiple testing with a two-stage approach describe in Benjamini and Hochberg (1995) (Supplementary Table S8). The best error rates are achieved with the highest number of samples.

CNN1d is the worst model across omics when trained on the whole training set. Convolutions are more suited for structured data, and even the incorporation of a structure in the data based on the chromosomal location is a constraint that limits the range of possible interactions. Only genes in the same convolution window interact with each other, which does not consider long-range interaction. The AttOmics model achieves better or similar performances than the best model that does not use self-attention across the different omics. For methylation data, the mean error rate is better than the GNN—CoExp approach. However, there is no statistical significance as the performances on the GNN are more variable across training. For gene expression data, similar error rates are achieved between MLP and GNN approaches.

Depending on the omics, non-deep-learning models do not perform equally. The SVM approach obtains similar performances for the methylation data to AttOmics, whereas XGBoost and RF achieve lower performances than the CNN1d model. XGBoost and AttOmics have the same performances for gene expression data. SVM and RF do not compete with other architectures and obtain one of the highest error rate. For the miRNA data, the best-performing methods are RF and XGBoost. The SVM approach obtains an error rate comparable to the deep-learning architecture, and there are no differences between the different deep-learning models (Supplementary Table S6).

We do not observe a difference in terms of performances between the different grouping strategies. For methylation data, random or clustering approaches give the same error rate. For gene expression data, GO and random grouping reach the same error rate, whereas the clustering approach has worse performances but still performs better than other deep learning approaches. The architecture based on the hallmarks grouping achieves performances similar to the MLP or GNN, whereas other grouping approaches improved the performance. The worst performance is probably due to this strategy’s implicit feature selection; only 4305 genes were used. A too-large selection of the number of features limits the potential for the model to learn the relevant interactions.

Since, in real-world applications, datasets are much smaller than the TCGA dataset, it is particularly interesting to analyse the performances of models trained from small training sets. Reducing the number of training examples affects model performance adversely, as a limited training database hinders the capacity of the model to extract hidden information during training. The performances vary similarly between the different grouping strategies when reducing the number of parameters. When training with the lowest number of examples, we can identify different sets of architectures. For mRNA, the MLP has the worst performance. All architectures incorporating a structure in the data (CNN and GNN) achieve similar performances. AttOmics architecture performs best and outperforms non-deep-learning methods with small training sets for gene expression and methylation data.

Only the hallmarks grouping strategy has worse performances than the other grouping strategies. We note that for the GNN architecture, the performances standard deviation across different training with the same number of training varies more than AttOmics architecture. This suggests that the GNN architecture depends on the selected training examples, whereas AttOmics models are less sensitive to this issue.

Other classification metrics were improved, such as the f1-score (Supplementary Fig. S4 and Table S7). The improvement is more significant when the number of training examples is limited.

The size of a neural network, i.e. its number of parameters to fit, has a strong impact on its performances and required hardware resources (memory, computing time). AttOmics architecture reduces the number of parameters compared to the CNN architecture or MLP and achieves similar or better performances (Supplementary Table S6). Due to the high number of features in the omics profile, most MLP parameters are within the first layer. However, the total number of parameters of a model is limited by the available hardware. One way to reduce the number of parameters would be to select features, but as explained earlier, this may lead to a loss of relevant information. Another approach would be to reduce the dimension of the first layer, therefore limiting the range of the possible number of neurons in the first hidden layer to meet the memory constraints. Limiting the first projection’s space leads to an extensive compression of the omics profile. With self-attention, we can increase the dimension of the projection by reducing the number of parameters and compressing the omics profile more gradually. For instance, for the mRNA clustering approach with self-attention, the profile is projected into a 43 280-dimensional space with only 125 million parameters, whereas projecting into the same dimension would require 2.5 billion parameters with an MLP.

As self-attention is known to have a quadratic complexity, we explored its impact on the memory usage of the model and the runtime to obtain prediction from the test set (Supplementary Fig. S7 and Table S9). During inference on the test set, there is a 45% increase in memory consumption with the MLP. With AttOmics, memory overhead range from 65% for the largest model to 250% for the smallest model. The runtime increases with AttOmics compared to the MLP but stays in the milliseconds’ range and is at least an order of magnitude better than the non-deep-learning methods.

A similar study is performed for prognosis prediction. The evolution of the C-Index according to the number of training examples is presented in Supplementary Fig. S6. All models obtain similar performance on this task. We note that the AttOmics architecture is more stable as it achieves a similar C-Index as the best state-of-the-art approach in different omics. Indeed, for DNAm data, AttOmics has a similar C-Index to the CNN architecture. For mRNA data, AttOmics has similar performances to the GNN. For miRNA data, the GNN architecture outperforms all other architectures.

To conclude these results, incorporating self-attention in the architecture allows having a softer compression of the features, improving the representation of the data and, therefore, the performances.

AttOmics works on methylation data (DNAm) and gene expression data (mRNA) but not on miRNA expression data. For miRNA data, the best performances were obtained with non-deep-learning approaches. There is some uncertainty when using biologically motivated groups, as the domain knowledge constantly evolves. When using biologically aware groups, the model is limited to only considering a subset of the possible feature interactions. Random or clustering grouping can detect relevant interactions not yet included in the biological knowledge. Future work will investigate different biological knowledge. Nevertheless, in this article, one can prefer biologically motivated groups that are more interpretable, although the other grouping strategies give slightly better results. This reflects the well-known trade-off between performance and interpretability in machine learning (Linardatos et al. 2021).

### 5.3 Attention map interpretation

**Figure 4.**
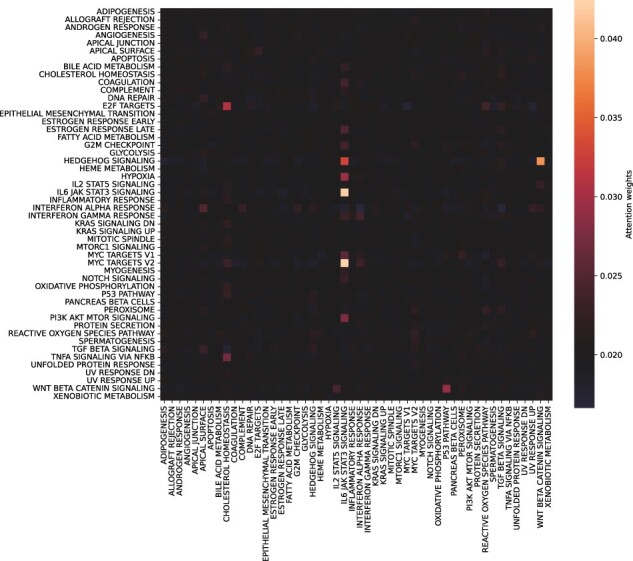
Attention map visualization for the TCGA-CESC class obtained after applying the hallmarks grouping on mRNA.

One advantage of using self-attention in the model is the ability to visualize the learned interactions (Fig. 4). The attention map corresponds to the attention weights average across patients with the same phenotype. The learned interactions are different across cancer (Supplementary Figs S9 and S10), suggesting that the model learns interactions specific to each cancer.

The self-attention mechanism used in the architecture was not designed to be an interpretability tool. However, it can provide information on the interaction learned by the model depending on the grouping strategy. We used GO slim or MSigDB hallmarks as a biological knowledge-aware grouping strategy. The terms used in GO slim represent general BPs; the high level of the terms makes it difficult to link the detected interaction with the phenotype. On the contrary, MSigDB hallmarks focuses on important and specific BPs and are, therefore, easier to interpret.

With the hallmarks grouping, we identified interactions between well-known pathways in cervical cancer: Wnt signaling, HedgeHog signaling, and JAK/STAT signaling (Fig. 4). STAT proteins play a role in the development of cervical cancer (Gutiérrez-Hoya and Soto-Cruz 2020). The inactivation of the Wnt pathway is known to promote cell growth in cervical cancer (Yang et al. 2018). It has been shown that HedgeHog pathway components are expressed in cervical cancer cells and are involved in cell proliferation (Samarzija and Beard 2012). It has also been shown that there is a crosstalk between Wnt and HedgeHog pathways, which are known to be involved in chemo-resistance in cervical cancer (Kumar et al. 2021).

Interestingly, we identified similar interactions learned by the model between the GO slim and hallmarks grouping strategies. For BLCA cancer, a model based on GO slim identified multiple interactions involved in the inflammatory response (GO : 0006954). In the model based on hallmarks, the inflammatory response group is also identified as involved in different interactions (Supplementary Fig. S11). The group composition between the two strategies is different, with an overlap of 29%.

The model can handle any grouping, handcrafted groups, or based on a different knowledge source that could be used to improve the information contained in an attention map.

## 6 Conclusion

In this article, we propose AttOmics, a novel deep-learning architecture for personalized medicine. AttOmics leverages self-attention to capture feature interactions specific to each patient. Features were grouped before applying self-attention on high dimensional vectors, such as omics profile. With this approach, we can reduce the number of parameters compared to an MLP with a similar dimension while accurately predicting the type of cancer. The self-attention also allows the visualization of the learned interactions to understand the model better. AttOmics is the only architecture consistently performing well on different omics data.

In future works, we will explore the use of linear approximation of self-attention maps for high-dimensional vectors. For example, to reduce the memory footprint of self-attention, new algorithms have been proposed to compute self-attention sequentially on chunks of queries (Rabe and Staats 2021). Different approximations have also been proposed like sparse attention (Child et al. 2021), which limits what each element can attend to, or Nyströmformer (Xiong et al. 2021), which computes attention using a modified Nyström approximation with linear complexity. Those new self-attention formulations could help its application on omics profiles.

In this study, the different omics profiles were studied individually. Considering the different omics profiles in a unique model could improve the prediction performances by exploiting the complementary information between the different omics profiles. The attention mechanism has proven to combine multiple modalities within a joint representation effectively. In our subsequent work, we will use attention-based multimodal deep-learning models to integrate the different omics data into the same model. Therefore a fusion of the different omics profiles with the attention mechanism could be computed directly onto the hidden layers of the model.

## Supplementary Material

btad232_Supplementary_Data
