# Supplementary material for: AttOmics: attention-based architecture for diagnosis and prognosis from omics data
Source: Bioinformatics. 2023 Jun 30;39(Suppl 1):i94–i102. doi: 10.1093/bioinformatics/btad232 (PMC10311315; doi:10.1093/bioinformatics/btad232)
Supplement: btad232_Supplementary_Data [file btad232_supplementary_data.pdf]

Supplementary materials: AttOmics: Attention-based architecture for diagnosis and prognosis from Omics data

Aurélien Beaude [1,2,*], Milad Rafiee Vahid [3], Franck Augé [2], Farida Zehraoui [1] and Blaise Hanczar [1,*]

[1]Université Paris-Saclay, Univ Evry, IBISC, 91020, Evry-Courcouronnes, France

[2]Sanofi R&D Data and Data Science, Artificial Intelligence & Deep Analytics, Omics Data Science, 1, Av Pierre Brossolette 91385, Chilly-Mazarin, France and

[3]Sanofi R&D Data and Data Science, Artificial Intelligence & Deep Analytics, Omics Data Science, 450 Water Street, Cambridge, MA, 02142 USA.

Table S1. Distribution of the samples across cancer and splits

Cancer	Train	Validation	Test	Total
BRCA	740	159	159	1058
UCEC	368	79	80	527
LGG	366	79	79	524
KIRC	355	76	77	508
THCA	354	76	76	506
LUAD	353	76	76	505
HNSC	344	74	74	492
PRAD	342	73	74	489
LUSC	331	71	71	473
SKCM	313	67	68	448
COAD	303	65	66	434
BLCA	282	60	61	403
OV	261	56	57	374
STAD	259	55	56	370
LIHC	257	55	56	368
Normal	252	54	55	361
CESC	205	44	44	293
KIRP	200	43	44	287
SARC	182	39	39	260
LAML	67	15	15	97
Total	6134	1316	1327	8777

Table S2. Graph dimension for the GCN model

Graph	Omics	Nodes	Edges
PPI	mRNA	15985	234984
CoExp	mRNA	17951	400632
	miRNA	314	1051
	DNAm	8360	390455

Table S3. Tested values of the hyper-parameters for the different architectures.

Model	Hyper-parameter	Tested values
MLP	Nb layers	[|1, 20|]
	Reduction ratio	[0.1, 0.9]
	Dropout	[0,1]
	Batch norm.	True/False
CNN1d	Kernel size	2,4,8
	Pooling size	2,4
	Num channels	32, 64, 128
GNN	Kernel size	X, 2, 4, 8
	Num channels	1, 16, 32
	Readout	concatenate, max, mean
SVM	C	0.1, 1, 10, 100, 1000
	gamma	1, 0.1, 0.01, 0.001, 0.0001
	Kernel	rbf, linear, poly, sigmoid
Random Forest	n_estimators	[1, 100]
	max_features	auto, sqrt
	max_depth	[3, 20]
	min_sample_splits	2, 5, 10, 15, 20, 25, 30, 50
	min_samples_leaf	1, 2, 4, 6, 8, 10, 20, 30, 50
	bootstrap	True/False
XGBoost	n_estimators	[1, 100]
	max_depth	3, 4, 5, 6, 8, 10, 12, 15
	gamma	0.0, 0.1, 0.2 , 0.3, 0.4
	min_child_weight	1, 3, 5, 7
	colsample_bytree	0.3, 0.4, 0.5 , 0.7
	learning_rate	0.05,0.10,0.15,0.20,0.25,0.30

Table S4. Selected hyper-parameters for the AttOmics architecture.

Grouping	Parameter	mRNA	DNAm	miRNA
Random	# group	20	10	5
	# blocks	4	3	2
	# heads	1	1	1
	group size	2164, 1459, 754, 50	1552, 826, 100	201, 80
Clustering	# group	20	20	10
	# blocks	4	3	1
	# heads	1	1	1
	group size	2164, 1459, 754, 50	776, 413, 50	50
GO	# group	46		
	# blocks	3		
	# heads	1		
	group size	200, 105, 10		

Table S5. Architecture used for the models. X not applicable. For the GCN model a - kernel means convolution from spatial space, and > 0 means convolution in the spectral space

Model	Parameter	mRNA	DNAm	miRNA
MLP	hidden dim	5739, 646	13675, 4103	807, 485
	dropout	0	0	0
	batch norm	True	True	True
CNN - 1D	kernel	4	2	2
	pooling	4	2	2
	# channels	64	64	64
	stride	1	1	1
GNN - PPI	# channels	1	X	X
	kernel	2	X	X
	readout	concat	X	X
GNN - CoExp	# channels	1	1	1
	kernel	-	-	2
	readout	concat	concat	concat
SVM	kernel	rbf	rbf	linear
	gamma	0.0001	0.0001	1
	C	100	1000	0.1
RF	n_estimators	95	88	99
	min_samples	10	2	5
	min_samples_leaf	1	1	1
	max_features	sqrt	auto	sqrt
	max_depth	18	None	16
	bootstrap	False	False	False
XGBoost	n_estimators	88	18	66
	min_child_weight	7	7	7
	max_depth	15	4	15
	learning_rate	0.15	0.25	0.15
	gamma	0	0.2	0.4
	colsample_bytree	0.5	0.5	0.5

Table S6. Models' sizes and their associated performances.

Omics	Model	# of parameters	Error-Rate	C-Index
	AttOmics - Clustering	28M	0.034 ± 0.002	0.722 ± 0.010
	AttOmics - Random	62M	0.034 ± 0.001	0.721 ± 0.005
DNAm	CNN1d	93M	0.042 ± 0.001	0.720 ± 0.008
	MLP	367M	0.039 ± 0.002	0.718 ± 0.002
	GNN - CoExp	8.6M	0.037 ± 0.003	0.708 ± 0.004
	SVM		0.034 ± 0.000	
	RF		0.050 ± 0.003	
	XGBoost		0.051 ± 0.000	
	AttOmics - Clustering	240M	0.043 ± 0.002	0.723 ± 0.006
	AttOmics - GO	28M	0.040 ± 0.002	0.721 ± 0.005
	AttOmics - Hallmarks	1.9M	0.046 ± 0.003	0.722 ± 0.009
mRNA	AttOmics - Random	240M	0.040 ± 0.002	0.726 ± 0.006
	CNN1d	470M	0.054 ± 0.001	0.714 ± 0.006
	MLP	333M	0.047 ± 0.002	0.717 ± 0.006
	GNN - CoExp	19M	0.047 ± 0.002	0.718 ± 0.012
	GNN - PPI	16M	0.047 ± 0.001	0.722 ± 0.003
	SVM		0.054 ± 0.000	
	RF		0.058 ± 0.001	
	XGBoost		0.035 ± 0.000	
	AttOmics - Clustering	0.22M	0.071 ± 0.004	0.679 ± 0.009
	AttOmics - Random	0.68M	0.077 ± 0.003	0.668 ± 0.002
miRNA	CNN1d	6.6M	0.120 ± 0.005	0.677 ± 0.004
	MLP	1.7M	0.077 ± 0.007	0.669 ± 0.011
	GNN - CoExp	0.37M	0.082 ± 0.012	0.697 ± 0.013
	SVM		0.034 ± 0.000	
	RF		0.050 ± 0.003	
	XGBoost		0.051 ± 0.000	

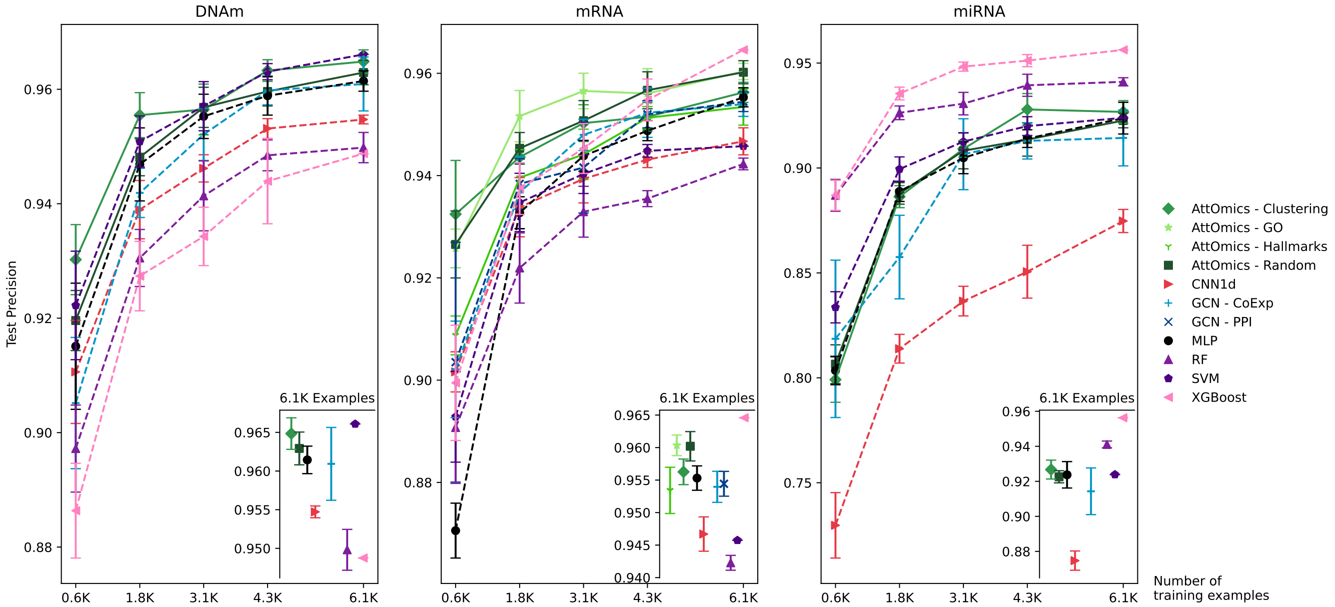

Figure S1: Precision on the test set according to the size of the training set. A * indicates a p-value < 0.05 with the best state-of-the-art method.

Table S7. Classification performances of the different models

		AUROC	Accuracy	F1-Score	Precision	Recall	Specificity
DNAm	AttOmics - Clustering	0.999 ± 0.000	0.966 ± 0.002	0.965 ± 0.002	0.965 ± 0.002	0.966 ± 0.002	0.998 ± 0.000
	AttOmics - Random	0.999 ± 0.000	0.966 ± 0.001	0.964 ± 0.002	0.963 ± 0.002	0.966 ± 0.001	0.998 ± 0.000
	CNN1d	0.998 ± 0.000	0.958 ± 0.001	0.956 ± 0.001	0.955 ± 0.001	0.958 ± 0.001	0.998 ± 0.000
	GCN - CoExp	0.999 ± 0.000	0.963 ± 0.003	0.962 ± 0.004	0.961 ± 0.005	0.963 ± 0.003	0.998 ± 0.000
	MLP	0.999 ± 0.000	0.961 ± 0.002	0.961 ± 0.002	0.961 ± 0.002	0.961 ± 0.002	0.998 ± 0.000
	RF	0.998 ± 0.000	0.950 ± 0.003	0.950 ± 0.003	0.950 ± 0.003	0.950 ± 0.003	0.997 ± 0.000
	SVM	0.998 ± 0.000	0.966 ± 0.000	0.966 ± 0.000	0.966 ± 0.000	0.966 ± 0.000	0.998 ± 0.000
	XGBoost	0.997 ± 0.000	0.949 ± 0.000	0.949 ± 0.000	0.949 ± 0.000	0.949 ± 0.000	0.997 ± 0.000
mRNA	AttOmics - Clustering	0.999 ± 0.000	0.957 ± 0.002	0.956 ± 0.002	0.956 ± 0.002	0.957 ± 0.002	0.998 ± 0.000
	AttOmics - GO	0.999 ± 0.000	0.960 ± 0.002	0.960 ± 0.002	0.960 ± 0.002	0.960 ± 0.002	0.998 ± 0.000
	AttOmics - Hallmarks	0.999 ± 0.000	0.954 ± 0.003	0.954 ± 0.004	0.953 ± 0.004	0.954 ± 0.003	0.998 ± 0.000
	AttOmics - Random	0.998 ± 0.000	0.960 ± 0.002	0.960 ± 0.002	0.960 ± 0.002	0.960 ± 0.002	0.998 ± 0.000
	CNN1d	0.997 ± 0.000	0.946 ± 0.003	0.946 ± 0.003	0.947 ± 0.003	0.946 ± 0.003	0.997 ± 0.000
	GCN - CoExp	0.999 ± 0.000	0.953 ± 0.002	0.953 ± 0.002	0.954 ± 0.002	0.953 ± 0.002	0.998 ± 0.000
	GCN - PPI	0.999 ± 0.000	0.953 ± 0.001	0.953 ± 0.001	0.954 ± 0.002	0.953 ± 0.001	0.998 ± 0.000
	MLP	0.997 ± 0.000	0.953 ± 0.002	0.954 ± 0.002	0.955 ± 0.002	0.953 ± 0.002	0.998 ± 0.000
	RF	0.998 ± 0.000	0.942 ± 0.001	0.942 ± 0.001	0.942 ± 0.001	0.942 ± 0.001	0.997 ± 0.000
	SVM	0.997 ± 0.000	0.946 ± 0.000	0.946 ± 0.000	0.946 ± 0.000	0.946 ± 0.000	0.997 ± 0.000
	XGBoost	0.999 ± 0.000	0.965 ± 0.000	0.965 ± 0.000	0.965 ± 0.000	0.965 ± 0.000	0.998 ± 0.000
miRNA	AttOmics - Clustering	0.997 ± 0.000	0.929 ± 0.004	0.927 ± 0.004	0.927 ± 0.005	0.929 ± 0.004	0.996 ± 0.000
	AttOmics - Random	0.996 ± 0.000	0.923 ± 0.003	0.922 ± 0.003	0.923 ± 0.003	0.923 ± 0.003	0.996 ± 0.000
	CNN1d	0.990 ± 0.000	0.880 ± 0.005	0.875 ± 0.005	0.875 ± 0.006	0.880 ± 0.005	0.994 ± 0.000
	GCN - CoExp	0.995 ± 0.001	0.918 ± 0.012	0.915 ± 0.013	0.914 ± 0.013	0.918 ± 0.012	0.996 ± 0.001
	MLP	0.995 ± 0.000	0.923 ± 0.007	0.923 ± 0.007	0.924 ± 0.008	0.923 ± 0.007	0.996 ± 0.000
	RF	0.997 ± 0.000	0.941 ± 0.002	0.941 ± 0.002	0.941 ± 0.002	0.941 ± 0.002	0.997 ± 0.000
	SVM	0.994 ± 0.000	0.924 ± 0.000	0.924 ± 0.000	0.924 ± 0.000	0.924 ± 0.000	0.996 ± 0.000
	XGBoost	0.998 ± 0.000	0.956 ± 0.000	0.956 ± 0.000	0.956 ± 0.000	0.956 ± 0.000	0.998 ± 0.000

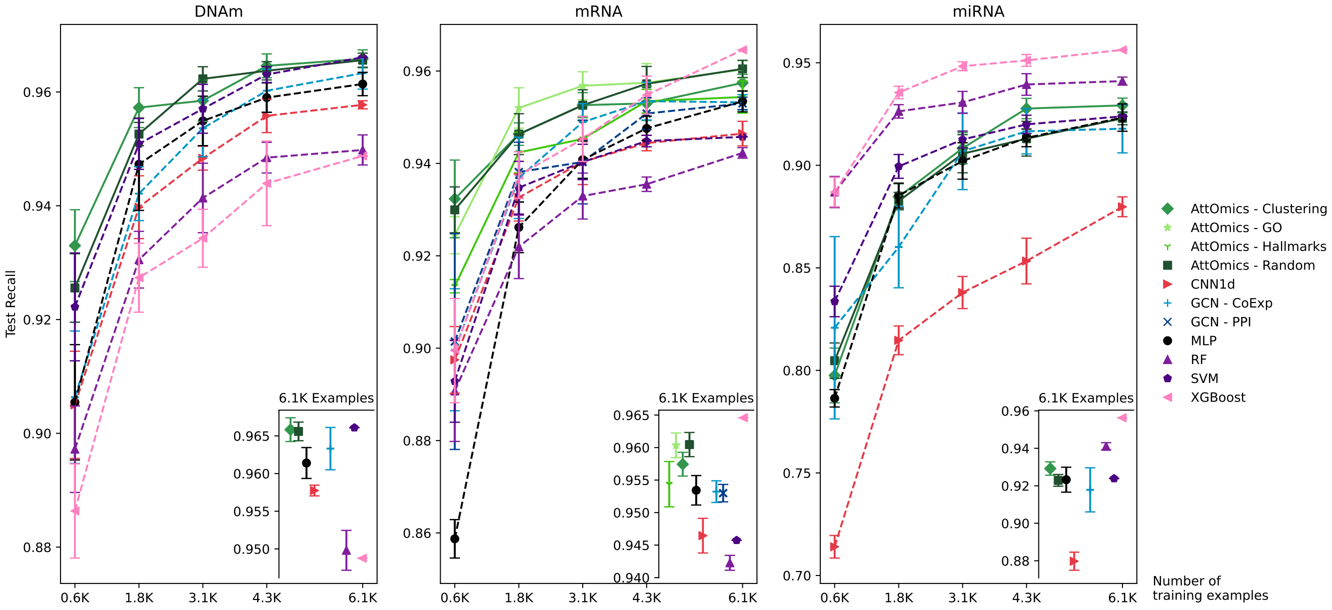

Figure S2: Recall on the test set according to the size of the training set. A * indicates a p-value < 0.05 with the best state-of-the-art method.

Table S8. P-value from the t-test on the error rate of the different models

omics	Model	AttOmics - GO	AttOmics - Hallmarks	AttOmics - Random	CNN1d	GCN - CoExp	MLP	RF	SVM	XGBoost	GCN - PPI
DNAm	AttOmics - Clustering	-	-	1.00	0.083	0.083	0.083	0.083	0.648	0.083	-
	AttOmics - Random	-	-	-	0.083	0.381	0.083	0.083	0.648	0.083	-
	CNN1d	-	-	-	-	0.083	0.083	0.083	0.083	0.083	-
	GCN - CoExp	-	-	-	-	-	0.510417	0.083	0.159091	0.083	-
	MLP	-	-	-	-	-	-	0.083	0.083	0.083	-
	RF	-	-	-	-	-	-	-	0.083	0.648148	-
	SVM	-	-	-	-	-	-	-	-	0.083	-
mRNA	AttOmics - Clustering	0.219	0.149	0.078	0.078	0.078	0.078	0.078	0.078	0.078	0.078
	AttOmics - GO	-	0.149	0.828	0.078	0.078	0.078	0.078	0.078	0.078	0.078
	AttOmics - Hallmarks	-	-	0.078	0.078	0.661	0.661	0.078	0.078	0.078	0.661
	AttOmics - Random	-	-	-	0.078	0.078	0.078	0.078	0.078	0.078	0.078
	CNN1d	-	-	-	-	0.078	0.078	0.078	0.828	0.078	0.078
	GCN - CoExp	-	-	-	-	-	0.501	0.078	0.078	0.078	0.661
	GCN - PPI	-	-	-	-	-	1.00	0.078	0.078	0.078	-
	MLP	-	-	-	-	-	-	0.078	0.078	0.078	-
	RF	-	-	-	-	-	-	-	0.078	0.078	-
	SVM	-	-	-	-	-	-	-	-	0.078	-
miRNA	AttOmics - Clustering	-	-	0.159	0.092	0.159	0.159	0.092	0.092	0.092	-
	AttOmics - Random	-	-	-	0.092	0.510	1.00	0.092	0.673	0.092	-
	CNN1d	-	-	-	-	0.092	0.092	0.092	0.092	0.092	-
	GCN - CoExp	-	-	-	-	-	0.673	0.092	0.510	0.092	-
	MLP	-	-	-	-	-	-	0.092	1.00	0.092	-
	RF	-	-	-	-	-	-	-	0.092	0.092	-
	SVM	-	-	-	-	-	-	-	-	0.092	-

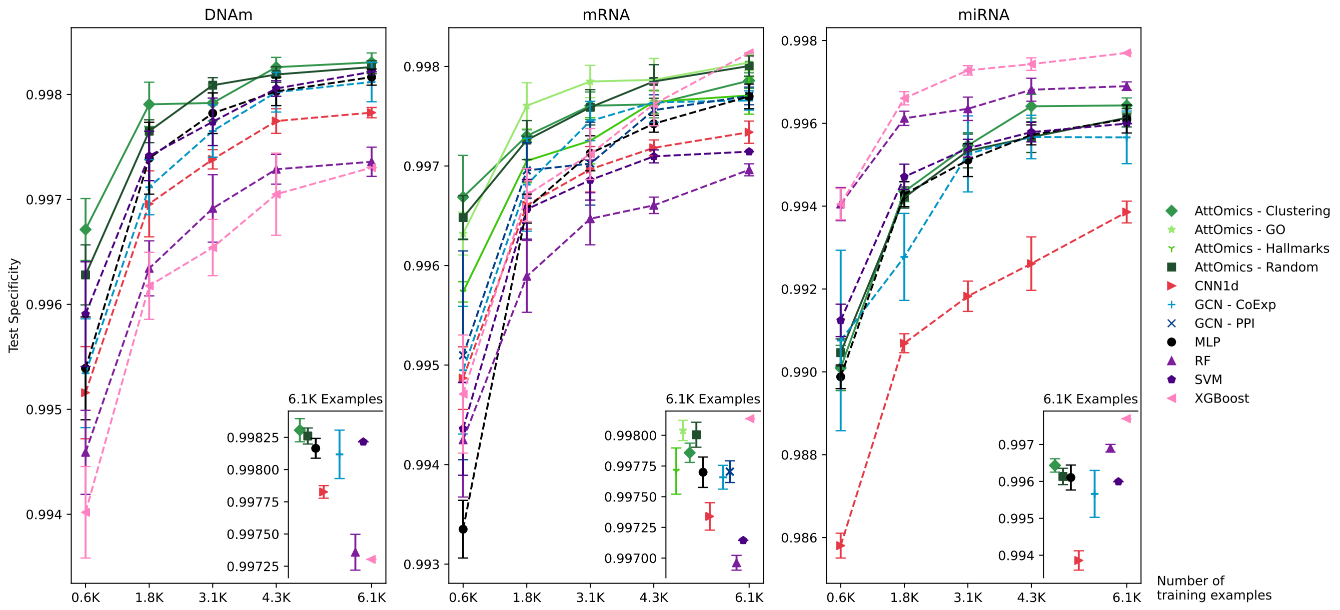

Figure S3: Specificity on the test set according to the size of the training set. A * indicates a p-value < 0.05 with the best state-of-the-art method.

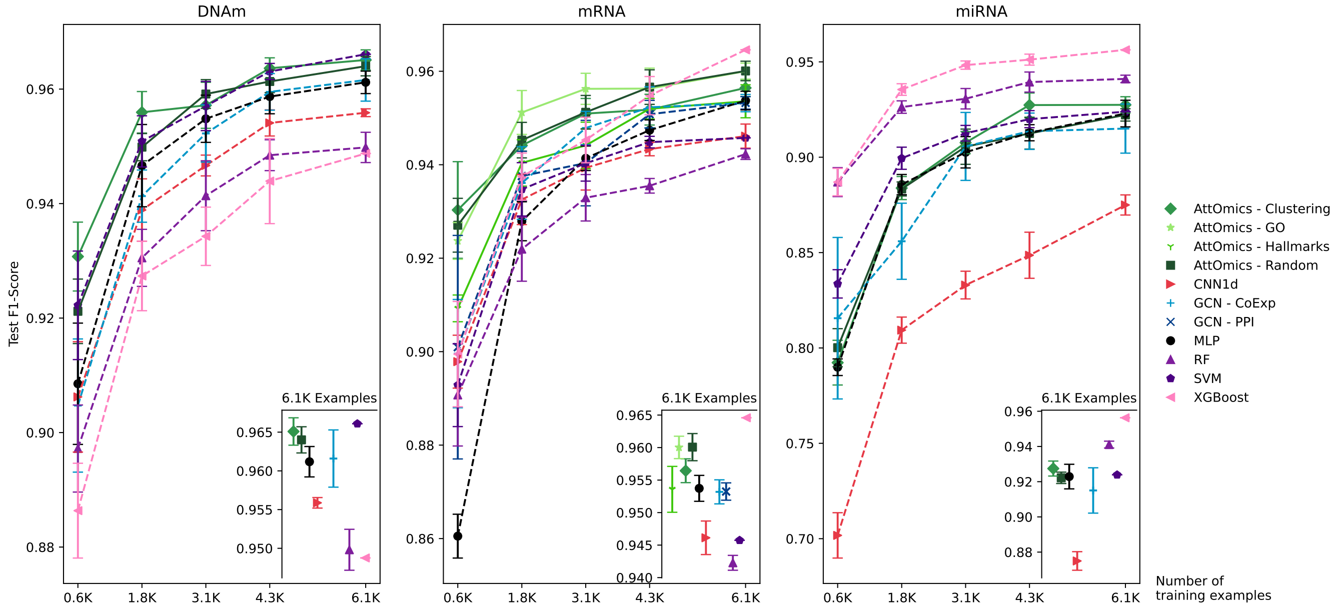

Figure S4: F1-Score on the test set according to the size of the training set. A * indicates a p-value < 0.05 with the best state-of-the-art method.

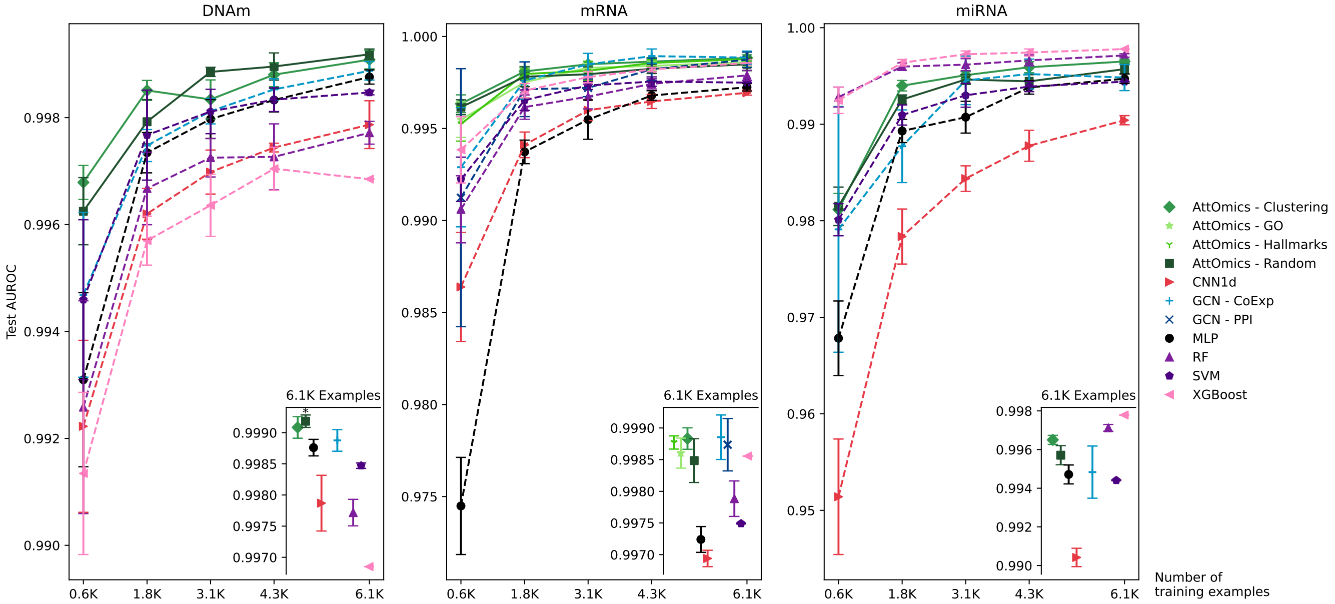

Figure S5: AUROC on the test set according to the size of the training set. A * indicates a p-value < 0.05 with the best state-of-the-art method.

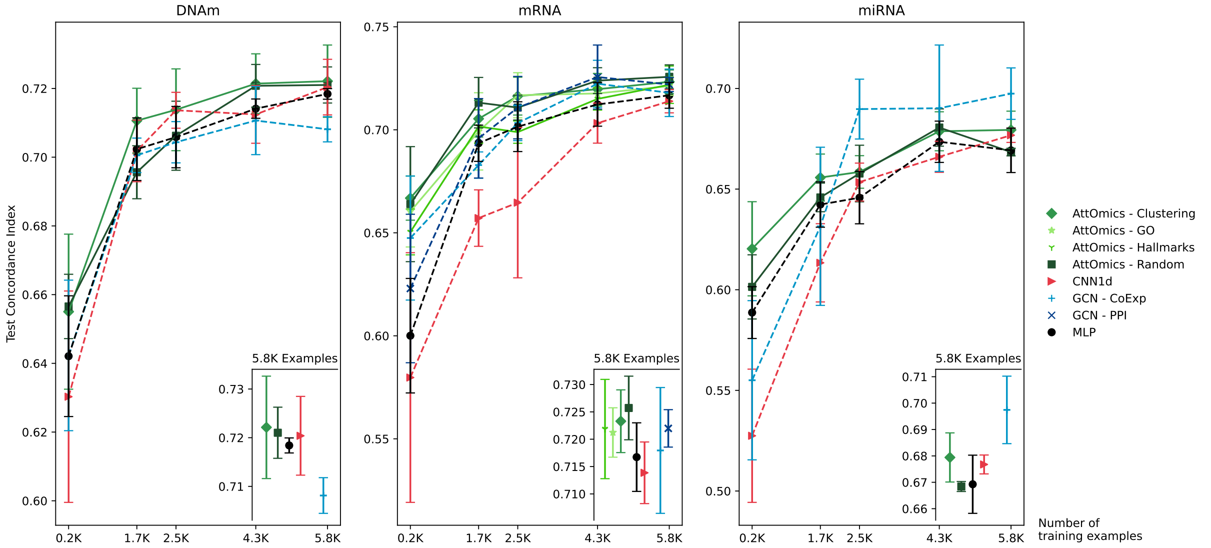

Figure S6: Concordance Index on the test set according to the size of the training set.

Table S9. Comparison of the time required to obtain predictions from the different models on the test set.

Omics	Model	Time (s)
DNAm	AttOmics – Clustering	0.006
	AttOmics – Random	0.004
	CNN1d	0.078
	GNN – CoExp	6.280
	MLP	0.001
	RF	0.046
	SVM	5.914
	XGBoost	0.019
mRNA	AttOmics – Clustering	0.008
	AttOmics – GO	0.028
	AttOmics – Hallmarks	0.005
	AttOmics – Random	0.008
	CNN1d	0.178
	GNN – CoExp	6.623
	GNN – PPI	2.564
	MLP	0.010
	RF	0.040
	SVM	12.850
	XGBoost	0.028
miRNA	AttOmics – Clustering	0.002
	AttOmics – Random	0.002
	CNN1d	0.006
	GNN – CoExp	0.326
	MLP	0.001
	RF	0.270
	SVM	20.868
	XGBoost	0.022

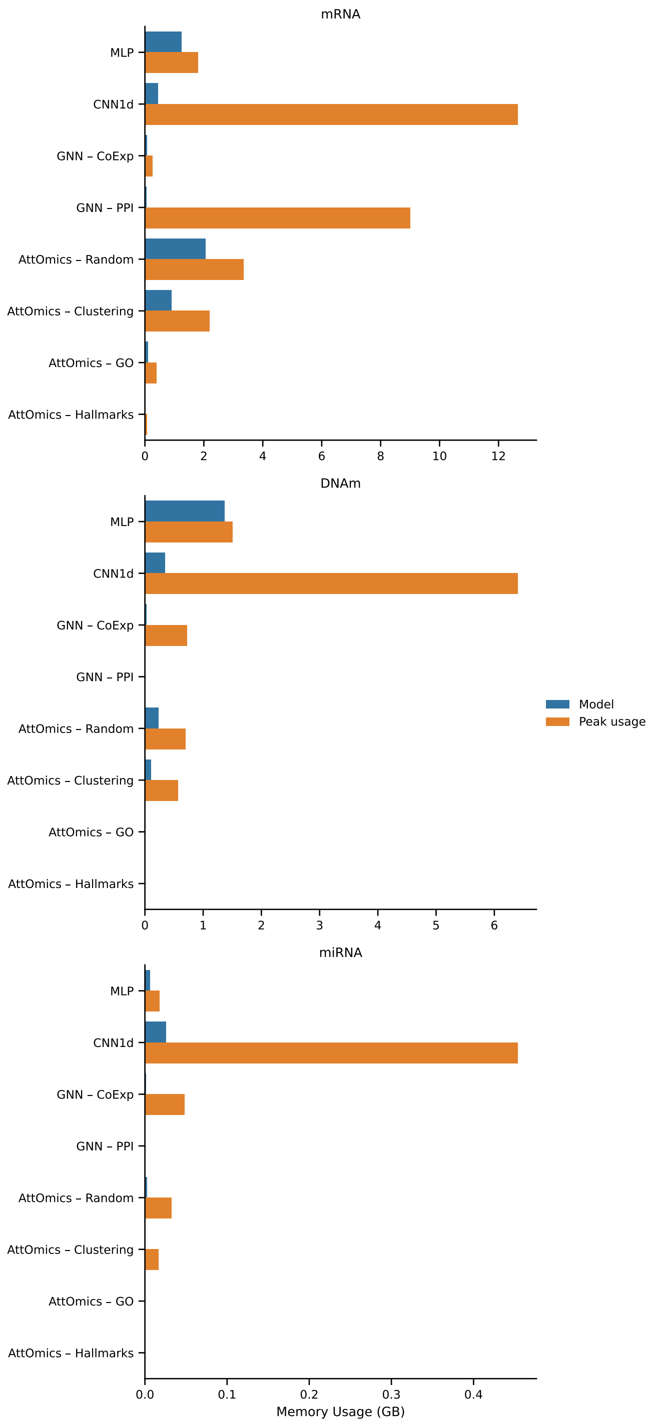

Figure S7: Comparison of the memory usage of the different deep-learning models and the peak of memory usage during inference on the test set.

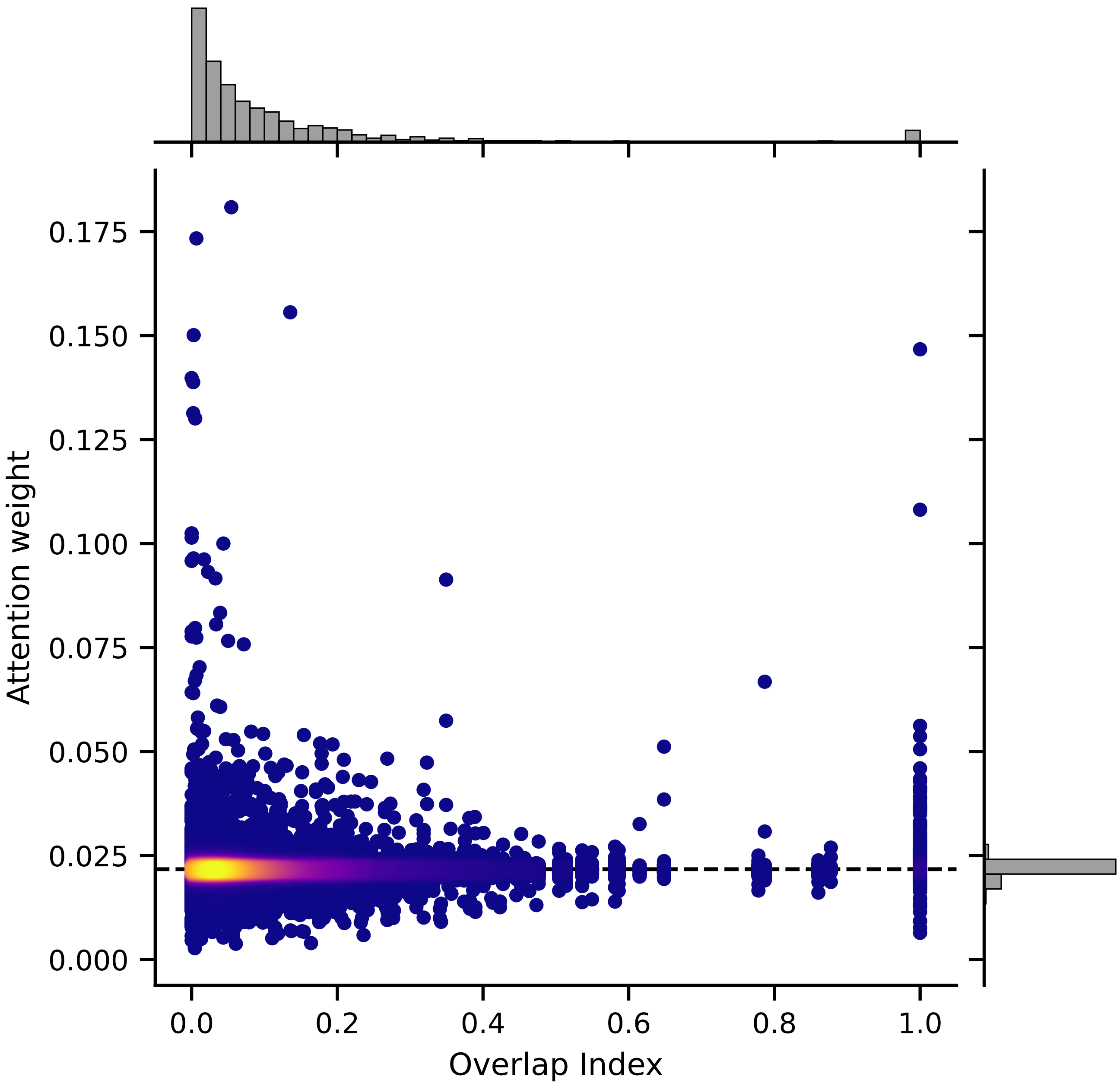

Figure S8: Attention weight and overlap index comparison for grouped based on GO. The dashed line correspond to the expected mean attention weight ($\frac{1}{n_{groups}}$). Points are colored based on the density of points.

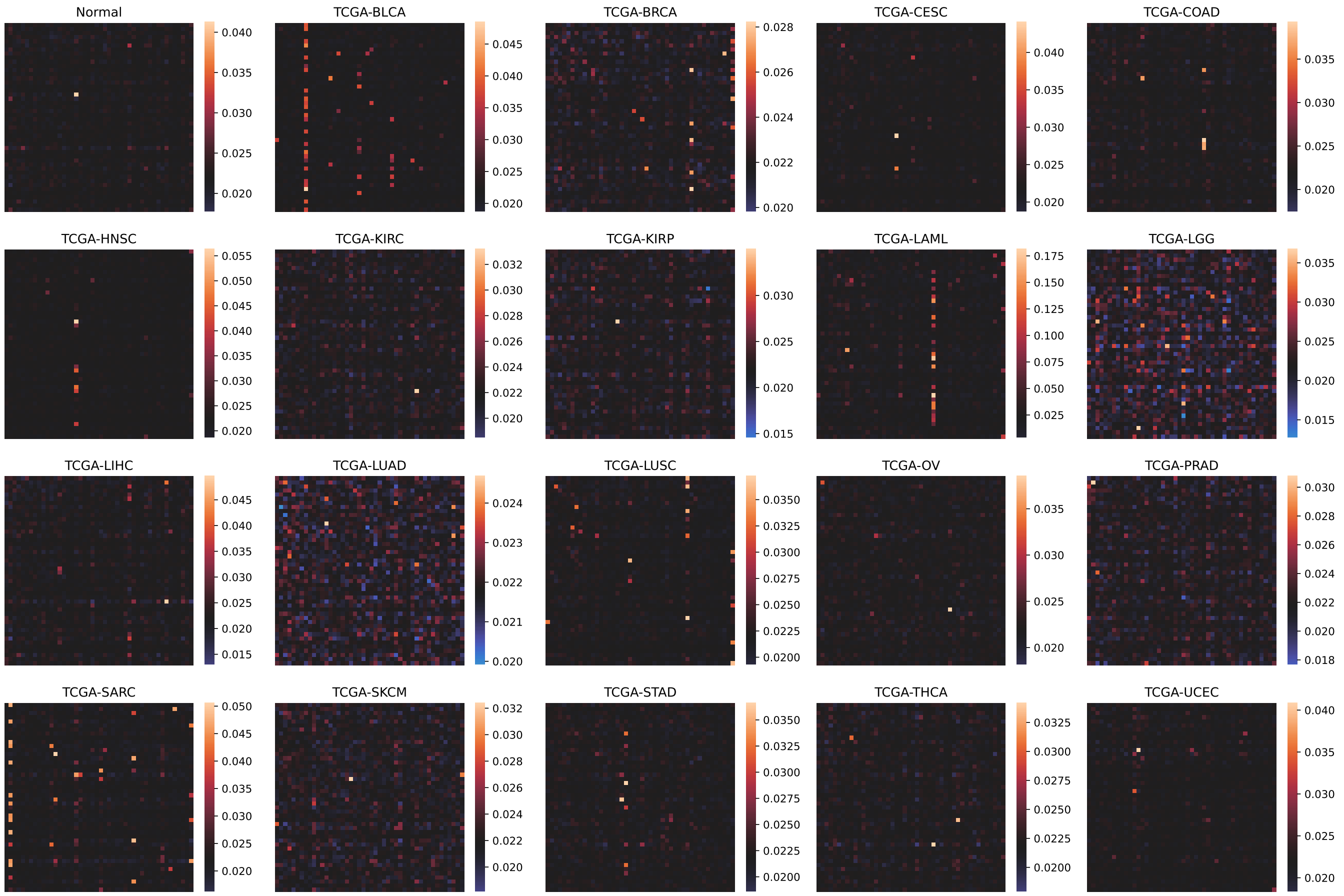

Figure S9: Attention map visualized per cancer for the first block when grouping with the gene ontology strategy. Attention map per cancer are obtained by taking the mean of the attention map of all patients with the corresponding cancer.

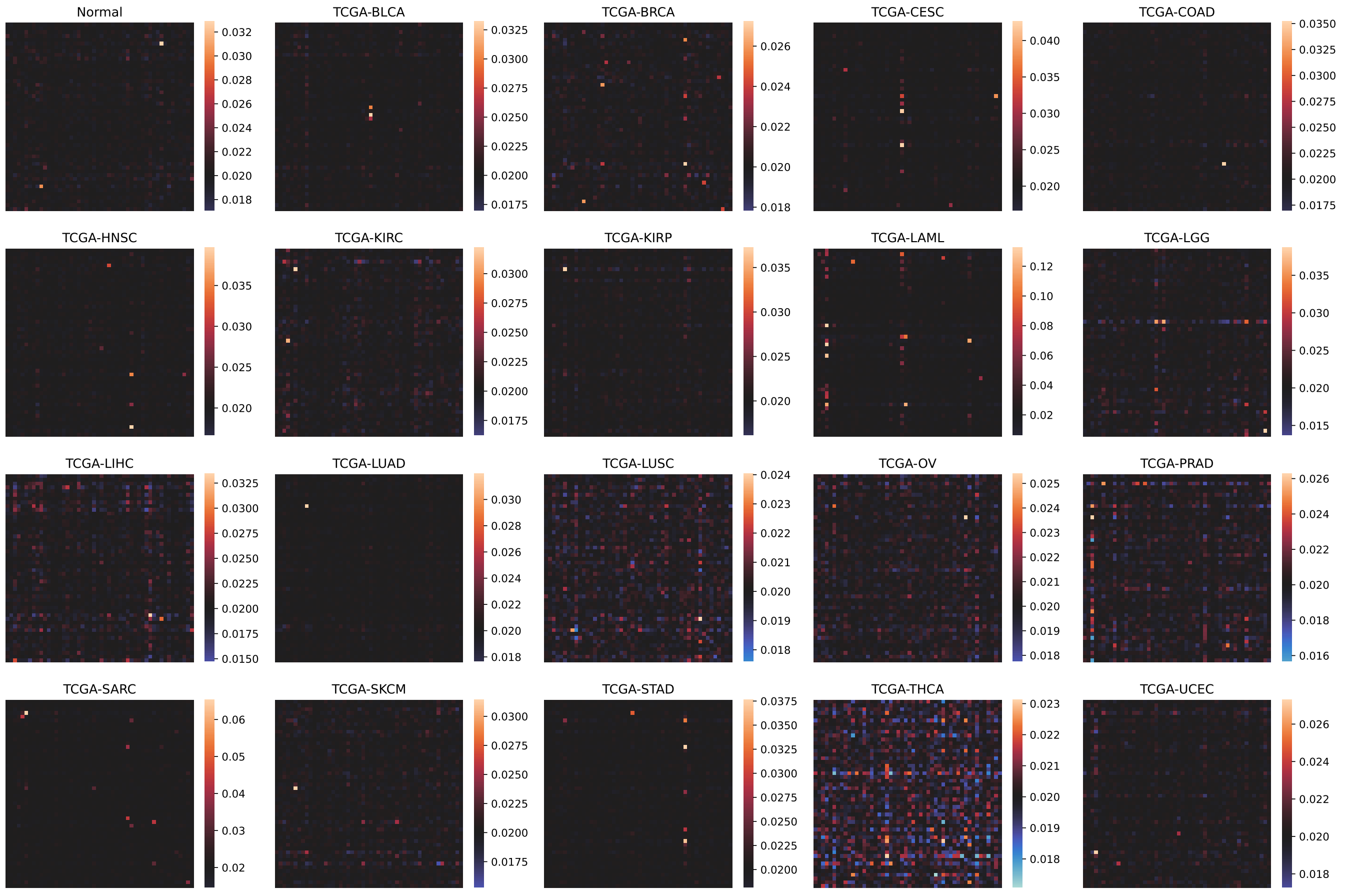

Figure S10: Attention map visualized per cancer for the first block when grouping with the hallmarks strategy. Attention map per cancer are obtained by taking the mean of the attention map of all patients with the corresponding cancer.

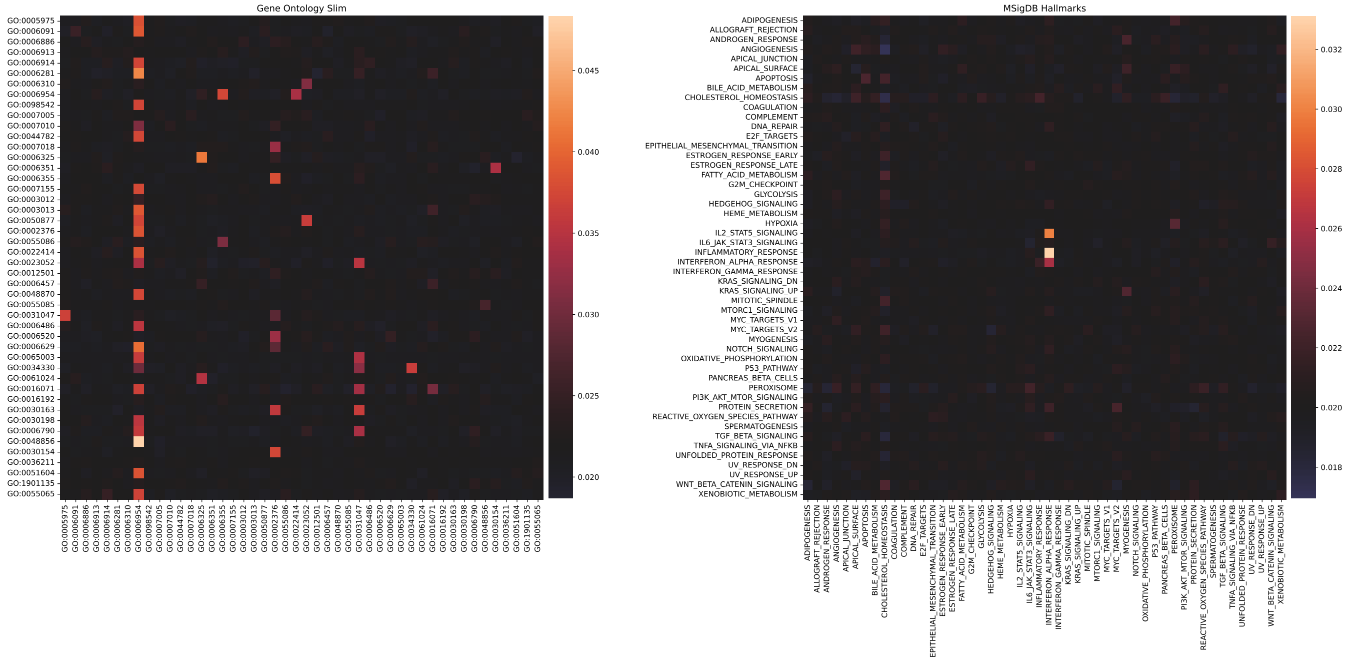

Figure S11: Comparison of the attention maps obtained with the gene ontology and the hallmarks grouping strategy. Both attention map highlights interaction involving an inflammatory response group.
